# Evaluation of the uniformity of UVA LED illumination on flat surfaces: Discrete ordinate method, single axis, and surface scanning radiometry

**DOI:** 10.1016/j.heliyon.2023.e16557

**Published:** 2023-05-20

**Authors:** Conor Reddick, Cintia Casado, Ken Reynolds, Simon Stanley, Cristina Pablos, Javier Marugán

**Affiliations:** a Department of Chemical and Environmental Technology, ESCET, Universidad Rey Juan Carlos, C/ Tulipán S/n, 28933 Móstoles, Madrid, Spain; b ProPhotonix IRL LTD, 3020 Euro Business Park, Little Island, Cork, T45 X211, Ireland

**Keywords:** LED Lamp, Radiometry, Spectroscopy, Simulation, Discrete ordinate method

## Abstract

Uniform illumination from UVA LED lamps is a crucial design characteristic for a range of industries including photocatalytic applications. In this work, radiometry and the discrete ordinate method (DOM) are used to determine the ideal target surface size and working distance from a UVA LED lamp for highly uniform illumination. Horizontal incident radiation and full surface incident radiation measurements were conducted using a scanning radiometry technique. It is shown that horizontal incident and full surface incident radiation measurements show good agreement for uniformity measurements over a range of working distances, with maximum uniformity (2.6% and 3.6% standard deviation respectively) over the measured range found at 15 mm working distance. DOM simulation results showed good agreement with radiometry for power and incident radiation measurements, whilst indicating a maximum uniformity at 20 mm working distance. These results demonstrate that DOM simulations can be used as a fast, low cost, and reliable indication of surface uniformity, peak surface irradiance, and power measurements in the design of UV lamps for industrial and academic applications.

## 1 Introduction

The advancement in UV LED technology since the beginning of the 21st century has seen the market grow to a capitalization of $1 billion in 2021 and continues to grow at a rapid rate [1]. The largest market segments include surface, air, and water disinfection (seeing a sharp increase in 2020 throughout the SARS-COV-2 pandemic), curing, horticulture, and detection [2]. In the case of UVA (315–400 nm) emitters, the viability of LED products in these markets has been driven by external quantum efficiencies as high as 50% [3], lamp lifetimes of 20,000 h [4], tunable wavelengths, and prices competitive with traditional UV lighting. The realization of high-powered UVA LED chips with a long lifetime equally relies on well-designed lamps, with thermal management systems and electronics able to control and protect the LED chips.

An emerging market for UVA LED is the treatment of water and wastewater by photocatalysis. Photocatalysis involves the irradiation of a photocatalytic material to reduce the activation energy of a chemical reaction. In the case of semiconductors such as TiO_2_, a photon of sufficient energy is absorbed by an electron in the valence band, promoting the electron to the conduction band. The promotion of this electron in turn creates an electron-hole pair. A hole in this context is a positively charged site on the semiconductor. Each of the electron and hole are free to partake in reactions to produce chemical species such as ·OH which is in turn able to oxidize chemicals and pathogens leading to their inactivation.

To make full use of a photocatalytic material within a treatment system, a high powered, UVA lamp with even illumination across the entire photocatalyst surface will improve homogeneity of photons arriving at the photocatalyst, which has been demonstrated to improve reactor efficiency in photocatalytic systems. Martín-Sómer et al. [5] used Ansys Fluent Discrete Ordinate Method (DOM) to simulate the radiation from a 40 LED lamp, an 8 LED lamp and a mercury fluorescent lamp within an annular reactor. Then, the annular reactor system was used for photo disinfection reactions. It was demonstrated that, for the same power output, the reaction rate was highest when using the mercury lamp, and lowest with the 8 LED lamp, the xenon-mercury lamp having the most homogeneous illumination field. In photocatalytic systems, it has been demonstrated that reaction rates are proportional to photon flux when photon flux values are relatively low, whilst at higher photon flux values, the reaction rate has an inverse square relationship with photon flux, becomes rαΦ12 [6]. This is due to increasing levels of electron-hole recombination as photon flux increases. The relationship between photon flux and reaction rates can help explain why even illumination is crucial to efficiency, as areas with higher-than-average illumination within the reactor will use photons less efficiently than areas of lower illumination. Electron-hole recombination can be reduced by connecting the photocatalyst to an external circuit, but due to the low positive voltage levels that can be applied to some photocatalytic materials caused by material instability, there will still be some recombination. Therefore, even illumination is likely to still be necessary in photoelectrocatalytic systems, although it is yet unproven.

Even illumination comes at a cost of total power delivered to the photocatalytic material, as a uniform irradiation pattern typically requires the lamp to be positioned outside of near-field as constructive interference in far-field leads to a smoother surface illumination [7], whilst peak incident radiation and power from a lamp decrease with working distance. Furthermore, high power output from LED is generated by increasing the forward current applied to the LED, which can cause a red shift of photons emitted from the lamp as the substrate temperature increases (i.e. lower energy photons). The uniformity from LED lamps can be improved by the use of reflector cups [8], diffuser plates [9], as well as the pattern shape [10] and spacing between LED [11].

Within literature, there are a range of methods used to measure the power and uniformity from a UV lamp. Gonio-radiometers allow for the measurement of irradiance across a single plane at a fixed radius from an LED lamp [12]. This technique allows for the measurement of irradiance through 180° at a fixed radius in front of the lamp as a means of quantifying the emittance profile from the LED lamp. Furthermore, radial data can be translated to a flat surface using ray tracing equations [12]. Typically, the profile from the gonio-radiometry is only along a single axis. Horizontal and vertical incident radiation profiles can qualitatively and quantitatively represent the homogeneity of illumination across a flat plane on a single axis to represent the uniformity through the center of a reactor [13] or through points of maximum incident radiation [14]. Photography techniques can also prove useful in qualitatively representing the homogeneity across a flat plane in two dimensions, whilst software packages such as Image J are able to analyse the warmth of light in an image [15].

Spatial uniformity measurements have also been conducted using a range of optics software packages, such as Zemax [16], LightTools [17], and TracePro [18]. Prior work has shown that these software packages can reliably represent the illumination patterns from a range of lamps and in turn, calculate the spatial uniformity. These software packages work based on ray tracing, where rays are simulated through a system, with the angle of reflection and refraction calculated at each surface encountered. The software package used in this work, ANSYS Fluent, uses discrete ordinates method (DOM), which solves the radiative transfer equation for a finite number of discrete solid angles, each associated with a vector direction fixed in a Cartesian system. The benefit of this technique for photocatalytic systems is that it can be coupled with surface chemistry and fluid dynamics in ANSYS Fluent to fully assess the characteristics of a photocatalytic system. It cannot be assumed that whilst ray tracing software packages have given reliable uniformity data in literature, DOM simulations will also give reliable data. Therefore, demonstrating that DOM is a suitable approach to calculate uniformity and power from a UVA LED lamp compared to radiometry measurements is a much-needed contribution to literature.

Sawyer et al. [17] evaluated the spatial uniformity on a flat surface from an LED ring lamp and fiber ring lamp for biomedical imaging applications using LightTools. It was found that the relationship between working distance and spatial uniformity for the fibre ring lamp followed an inverse square law thus suggesting the ideal working distance for highly uniform illumination is at a low working distance. The uniformity-working distance relationship for the LED ring lamp did not follow a similar trend, with the authors suggesting the ideal working distance for maximum uniformity existed outside of the range of their measurements, as uniformity increases with working distance.

This paper aims to validate a methodology to find a point of high spatial uniformity close to the target surface, where high power is also observed.

Analysis of spatial uniformity typically involves finding the maximum and minimum incident radiation values and finding nonuniformity from Eq. (1) [19]:

(1) $$Non\;uniformity=\frac{\mathrm{Max}-\mathrm{Min}}{\mathrm{Max}+\mathrm{Min}}\times 100$$

One study within literature attached a radiometer to a rail system, which was able to move the radiometer in a flat field in front of arrays of white LED as a method to assess the uniformity of radiance across the flat surface at a fixed distance from the lamp [20]. As the radiometer moved in the X and Y directions in front of the LED array, the radiometer collected irradiance measurements (mW m^−2^). As a statistical population is gathered using this method, variance was used to assess uniformity. The spatial uniformity was calculated using Eq. (2) [20].

(2) $$\varepsilon_{C}\left(p,\lambda \right)=100\times \left[\frac{DN_{C}\left(p,\lambda \right)}{DN_{C}\left(0,\lambda \right)}-1\right]$$

Where.

*ε*_*c*_*(p,λ)* is the standard deviation at position *p* and wavelength *λ,*
*DN*_*C*_*(p,λ)* the spectral irradiance at position p and wavelength λ, and
*DN*_*C*_*(0,λ*) the spectral irradiance at measurement center point and wavelength *λ.*

The radiometry technique has been demonstrated as especially useful in near-field irradiation because qualitative measurements such as surface plot images or horizontal incident radiation plots can show the individual LED irradiance, suggesting low homogeneity in lamps, whilst a statistical technique will better represent the population [20]. Similarly, variance may better represent uniformity from lamps with asymmetrical LED pitching, or where the beam shape and target surface are incongruous. Talone et al. [20] used Eq. (2) to represent irradiance measurement uniformity from the center point to other points in the X and Y direction in a single scan before calculating standard deviation. In this paper, Eq. (3) will be used to calculate standard deviation to compare between scans at various surface sizes and working distances between the lamp and detector.

(3) $${\%\;SD}_{I}=\frac{{SD}_{I}}{\bar{I}}\times 100$$

Where.

*%SD_I_* is the percentage standard deviation of radiometry data in a scan,
*Ῑ* the average surface incident radiation,
*SD_I_* the standard deviation of radiometry data in a scan.

This paper investigates the relationship between uniformity of irradiance from a UVA LED lamp and the total power delivered to a flat surface (64 cm^2^) using a scanning radiometry technique over a range of working distances. Furthermore, the study demonstrates that calculating variance from maximum horizontal incident radiation measurements give a good indication of uniformity compared to the full radiometry scan. The power, peak incident radiation, and uniformity measurements from radiometry are compared to results from Ansys Fluent Discrete Ordinate Method (DOM) simulations of the same lamp geometry. Finally, spectroscopy is used to measure the variance in wavelength and power output from LED, as well as their power loss and spectral shift against operating time. Measuring the individual spectrums of LED within the lamp demonstrates the role individual LED have on the overall uniformity from the lamp.

## 2 Experimental

### 2.1 UVA LED lamp

**Fig. 1:**
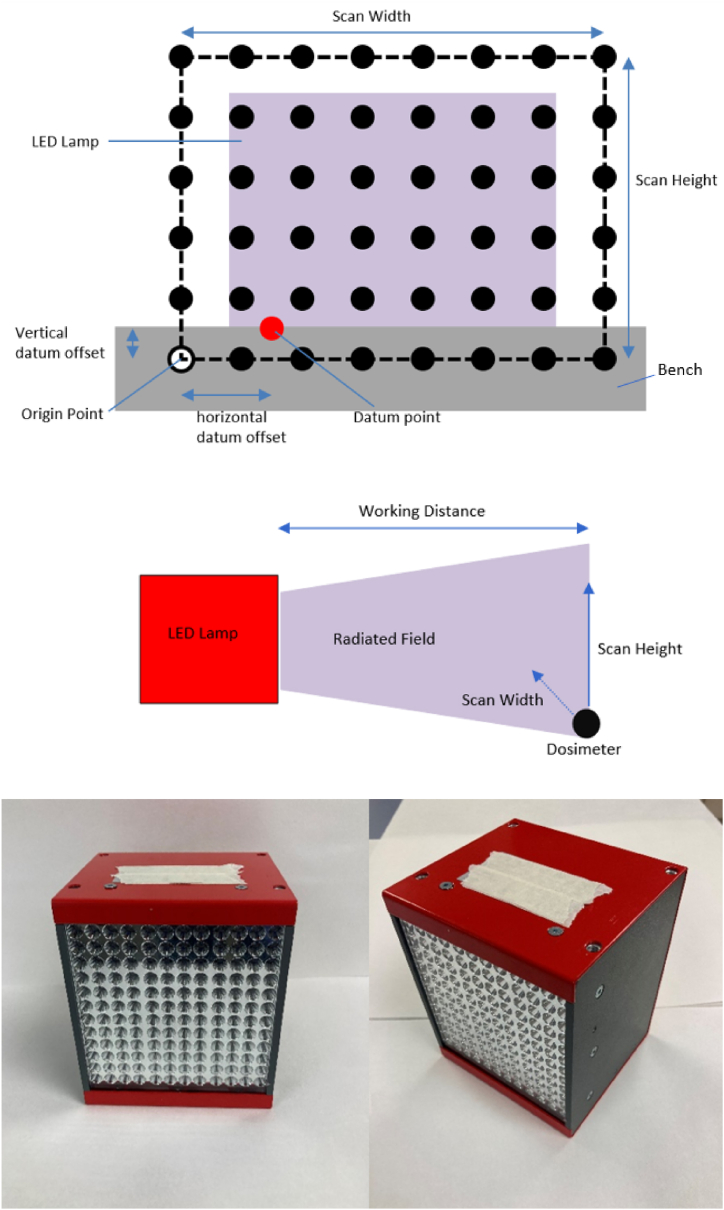
Top: Front view diagram of motor gantry. Middle: Side view diagram of motor gantry. Black dots in diagram represent the position of radiometer during scan. Bottom Left and Right: Pictures of UVA LED lamp used for radiometry experiments.

The lamp characterized in this paper contains 144 LED chips (365 nm ± 5 nm @ 25 °C, GaN based structure, operating temperature range −30 °C to +85 °C, maximum DC forward current 700 mA @ 25 °C, Seoul Viosys UV1000-36, 1.05 mm square [21]) arranged in a 12 by 12 square of approximate height and width 100 mm, with a pitch of 8 mm between LED. The LED substrate temperature is digitally monitored, with the lamp shutting off if reaching a high temperature. The lamp is cooled by a mounted fan (Sanyo Denki, 9G0624S101). The LED are split into three separately controllable channels of 42 LED where the distance between LED in neighboring channels is 9 mm. To improve uniformity of illumination across a flat surface and to minimize light lost due to high angle emittance from the LED, a block of polished aluminum, cone shaped reflector cups is positioned over the LED. Each reflector cup is 7 mm high, with a pitch angle of 36°. The LED are bare chip (i.e. do not have encapsulated lenses). The lamp was attached to an LED strobe controller (Smartek IPSC4r2, max current output per channel 1 A @ 30 V). The lamp was powered by a 10 A, 24 V power supply. Photos of the lamp are shown in Fig. 1.

### 2.2 Radiometer

Irradiance measurements were conducted using an Ophir PD-300 R M-8W dosimeter (spectral range 350–850 nm, irradiance range 0.2·10^−3^ – 8 W cm^−2^, damage threshold 50 W cm^−2^, operating temperature limit 70 °C, aperture size 8 mm, sample rate 500 s^−1^), with a cosine corrected PTFE diffuser (cosine correction factor accuracy 5%), connected to a power meter (Ophir StarBright). Spectral measurements of the LED used in the lamp verify that the peak begins at 353 nm, confirming no signal is out of range of the radiometer. Good agreement between an ideal cosine response and the measured angle of acceptance for this radiometer has been shown previously in literature [22]. The radiometer is calibrated over the full spectral range (350–850 nm), with the user able to access these calibrations by entering any wavelength value in the calibration range into the power meter. To measure the irradiance from the light source for all tests, a wavelength of 365 nm was selected. The full width half maximum of the LED is 10 nm which can be considered relatively narrow with an approximately Gaussian spectral shape. Given the near linear relationship between photon energy and wavelength in this range, coupled with the Gaussian shape of the LED spectra, there are roughly equal amounts of photons distributed symmetrically above and below 365 nm, meaning 365 nm can be taken as the average energy of all photons measured from the LED. A diagram of the photon energy and LED spectrum is shown in Fig. S1. The measurement uncertainty for all irradiance measurements is +1 W m^−2^. A scan averaging time of 1 s was used for all measurements.

### 2.3 Motor gantry

A motor gantry was used to measure irradiance across a flat plane parallel to the LED lamp's window. The radiometer is mounted to the motor gantry to collect data points across the flat plane. The motor gantry was powered by an Arduino nano unit and controlled from a LabView programme. All measurements were conducted in a black box to minimize the impact of stray or ambient light. As an additional measure, the radiometer was zeroed to remove any signal detected from ambient light before each measurement. The motor gantry performs a scan by taking a datum point, offsetting the origin point from the datum point in the horizontal and vertical direction at values set by the user, and then scanning a set number of columns across a total horizontal distance, whilst collecting an user selected number of measurements along the chosen scan height. In operation, the motor moves smoothly and at a consistent speed. Minimal vibrational movement is generated by the motor. The measurements were conducted in a room that is maintained at a constant temperature of 21 °C with no other sources of movement or noise present in the room. A diagram of the motor gantry used to measure irradiance is shown in Fig. 1. Images of the lamp, radiometer, and motor gantry within the black box are shown in Fig*.* S*2*.

### 2.4 Peak incident radiation vs working distance measurements

To ensure the peak incident radiation is captured, 25 mm × 25 mm scans were performed in front of the center of a large enough area to capture the peak incident radiation across all working distances. Irradiance data was collected every 2.5 mm in the x and y directions. A dwell time of 1 s was set in the LabView configuration time to allow for a static measurement of irradiance. Working distances between the lamp window and the radiometer were measured using a slip gauge at three points across the lamp window to ensure the lamp was positioned parallel to the motor gantry in the horizontal and vertical directions. Working distance measurements were taken between 10 and 60 mm. To minimize the effect of substrate temperature on the irradiance measurements, the order of tests was randomized. Working distance measurements were taken in triplicate. Table S1 in the supplementary material shows the settings used for each scan.

### 2.5 Incident radiation vs current measurements

Incident radiation – current measurements were taken at a working distance of 15 mm from the lamp. The working distance for these measurements was selected after the optimal working distance for uniformity was determined (Fig. 8). During measurements, the lamp was continuously on with 10 min given between readings to allow the irradiance output to reach a steady value. Since the applied current will affect the temperature of the substrate, the order of tests was randomized. Incident radiation – current measurements were taken in triplicate. Table S1 in the supplementary material shows the settings used for each scan, whilst Fig. S3 shows the results.

### 2.6 Single axis incident radiation measurements

A single set of irradiance points were captured horizontally through the center of the UVA LED lamp at working distances from 10 to 60 mm. A point was captured every 1 mm across a 150 mm scan width. Table S1 in the supplementary material shows the settings used for each scan.

### 2.7 Surface irradiance measurements

150 × 150 mm scans were performed to collect irradiance values across an area larger than the target surface. Irradiance data was collected every 2.5 mm in the x and every 5 mm in the y direction. Surface irradiance measurements were conducted at several working distances between 10 and 60 mm from the lamp. As the lamp operated at a continuous current through the experiments, a single warm up time of 1 h was allowed for the lamp to reach a steady power output.

### 2.8 Spectroscopy measurements

Spectroscopy measurements were conducted using an OceanOptics USB4000 Fibre Optic Spectrometer, a UV–Vis fibre (Ocean Insight, wavelength range 300–1100 nm), and a CC-3 Cosine Corrector (OceanInsight, opaline glass, wavelength range 350–1000 nm). The integration time was set to 10 ms to ensure saturation did not occur, with OceanView 1.6.7 software averaging 10 scans to display the wavelength spectrum. A background measurement was taken of the environment before turning on the lamp to eliminate interference from external light sources.

The spectrum of every LED on the lamp was measured to determine the average and range of peak wavelengths and power from the chips. The lamp was switched on for 60 min prior to measurements to reach a constant output power. The cosine corrector was positioned directly in front of each LED and in contact with the window of the lamp. Each channel of the lamp was provided with 250 mA for these measurements.

Spectral measurements of a single LED from the flood lamp were conducted to characterize the spectral shift over time as the lamp increased in temperature. Measurements were taken from t = 10 s up to t = 3600 s after the lamp was switched on. The cosine corrector was positioned directly in front of the LED and in contact with the quartz sheet of the lamp. The results from these tests are included in the supplementary material, Fig. S3.

### 2.9 Discrete ordinate method

The Radiative Transfer Equation (RTE) describes the conservation of radiative intensity in a direction of space.

(4) $$\frac{{dI}_{\lambda ,\underline{\Omega }}}{ds}={-\kappa }_{\lambda }I_{\lambda ,\underline{\Omega }}-\sigma_{\lambda }I_{\lambda ,\underline{\Omega }}+\frac{\sigma_{\lambda }}{4\pi }\int_{\Omega^{\prime }=4\pi }p\left(\underline{\Omega^{\prime }}\to \underline{\Omega }\right)I_{\lambda ,\underline{\Omega^{\prime }}}d\Omega^{\prime }$$

Where: $I_{\lambda ,\underline{\Omega }}$ = intensity of photons with wavelength λ, travelling in direction Ω

$p\left(\underline{\Omega^{\prime }}\to \underline{\Omega }\right)$ = scattering phase function

$\kappa_{\lambda }$ = volumetric absorption coefficient for a specific wavelength band

s = path length

$\sigma_{s}$ = scattering coefficient

σ = Stefan-Boltzmann constant (5.669 × 10^−8^ W/m^2^ -K^4^).

$\Omega^{\prime }$ = solid angle

**Fig. 2:**
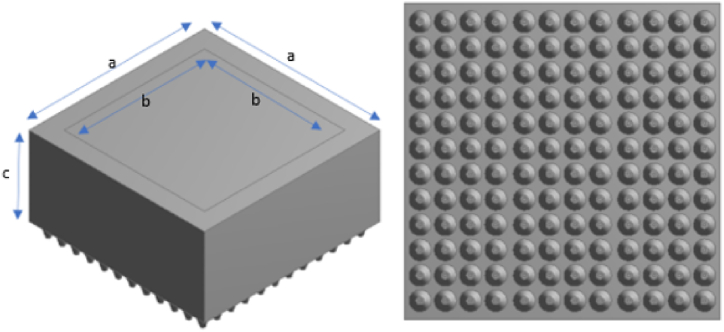
Left: Geometry of black box enclosure. A = 100 mm, b = 80 mm, c = working distance (mm). Right: UVA LED lamp geometry.

The RTE is solved using the commercial computational fluid dynamics package ANSYS Fluent. The geometry consisted of a UVA LED lamp and an enclosed black box to measure the surface incident radiation acting upon a target surface parallel to the emitting face of the LED in the lamp, shown in Fig. 2. To measure the surface incident radiation at various working distances from the lamp, the depth of the black box varied to match the desired working distance between the target surface and lamp.

Through mesh sensitivity analysis at the minimum working distance between the wall and lamp (10 mm), it was found that above 623,000 tetrahedral cells gave consistent area weighted average surface incident radiation values. The same cell sizing values (element size = 8·10^−4^ m, growth rate = 1.2, defeature size = 1·10^−5^ m, curvature minimum size = 1·10^−5^ m, minimum edge size = 1·10^−4^ m) were used for all meshes in the larger geometries. The number of cells on the target wall was kept constant.

To simulate the bare chip LED in the lamp, a beam angle of 120° was used [23]. The reflector cups used in the lamp are made of polished aluminum, with an internal emissivity of 0.1 and a diffuse fraction of 0.1. Temperature was set to 1 K to reduce computational time spent solving RTE by disregarding the energy term of equation. A direct irradiation value of 1560 W m^−2^ was set for each LED, which is equal to the measured irradiance by radiometry at 0 mm for an applied current of 250 mA/channel, as found by regression analysis of the irradiance vs working distance radiometry data. All other walls are modelled as black bodies. The convergence criteria for intensity was set to 10^−6^ and the solution method for the discrete ordinate equation was set to second order upwind.

## 3 Results and discussion

### 3.1 Selection of surface size

**Fig. 3:**
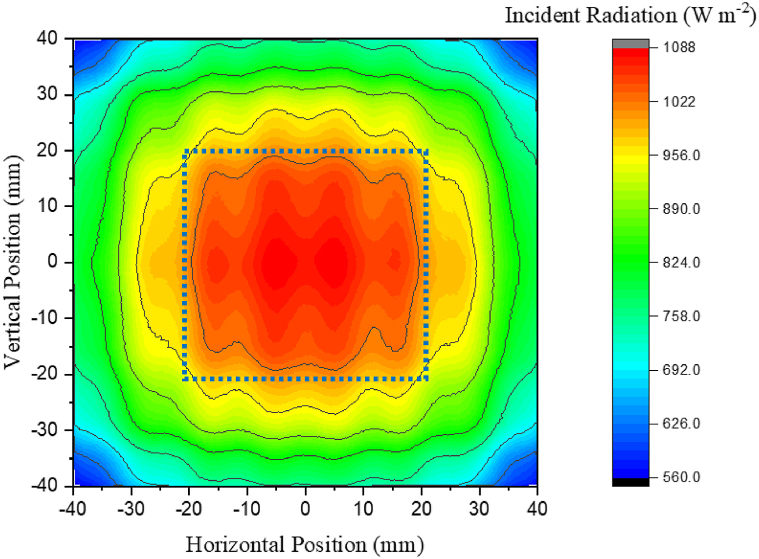
Example of tradeoff between total photons acting on a surface and uniformity of illumination. Surface within blue dashed line experiences highly uniform illumination but at the expense of not capturing the photons outside this area.

Before scanning radiometry and DOM simulations can be conducted, it is necessary to select a target surface size. The selection of an ideal surface size illuminated by a lamp requires some trade-off between the uniformity of illumination on the target surface and the fraction of photons emitted from the lamp that reach the target surface. Capturing a large proportion of the radiation emitted from a lamp would require a target surface larger than the lamp face but at the expense of uniformity, as the incident radiation of light will become low at the corners and edges. A smaller surface will see greater uniformity but a drop in power due to losses of light, highlighting the trade-off between total power and uniformity of light arriving at the surface, as shown in Fig. 3. The distance between the lamp and target surface also plays a role, as surfaces smaller than the lamp face will capture more radiation when placed close to the lamp. For surfaces much larger than the lamp face, the radiation emitted from the lamp reaches the target surface but in varying uniformity with working distance.

**Fig. 4:**
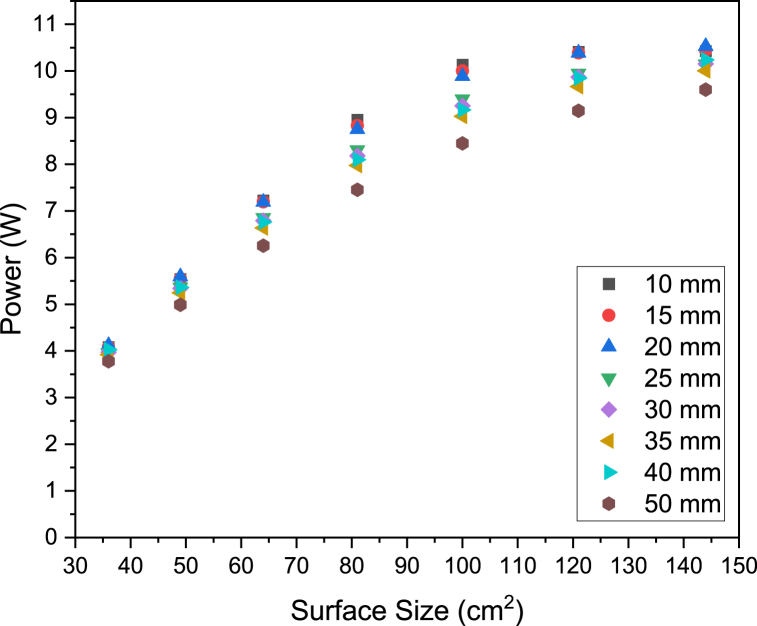
Power reaching target surfaces of different sizes at a range of distances between the lamp and surface (working distance).

**Fig. 5:**
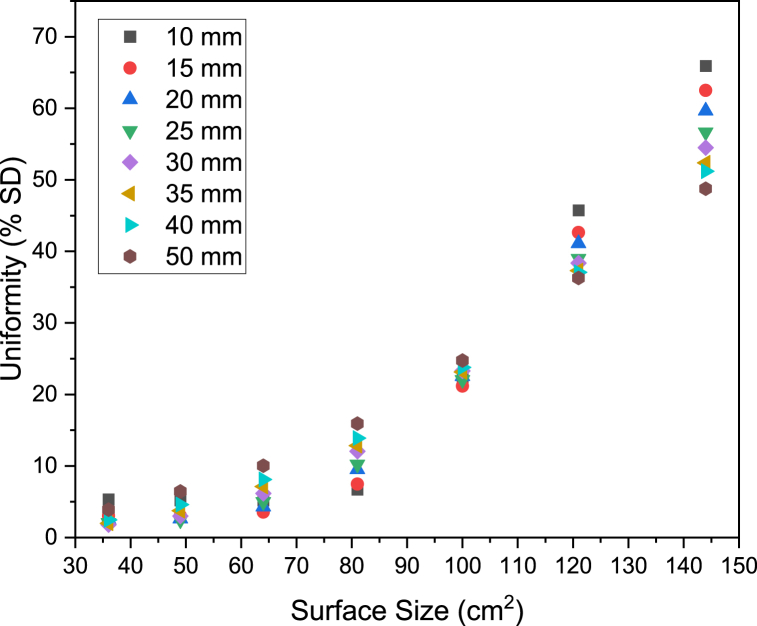
Percentage standard deviation of surface incident radiation reaching target surfaces of different sizes at a range of distances between the lamp and surface (working distance).

Fig. 4 shows how the power measured by radiometry is related to the surface size. The power delivered to the target surface increases linearly from 36 to 100 cm^2^. At target surface sizes above 100 cm^2^, the power plateaus. The emitting face of the lamp is closest in size to the 100 cm^2^ target face and given the reflector cups fitted on the LED are intended to emit radiation at a low angle, it would be expected that surfaces larger than 100 cm^2^ would not capture much more radiation. Fig. 5 shows the uniformity of light, measured by percentage standard deviation from the average incident radiation value, for a range of target surface sizes. The standard deviation calculated with Eq. (3) remains below 10% for surfaces below 64 cm^2^ at all working distances, whilst for surface sizes above 64 cm^2^, the uniformity drops significantly.

Fig. 5 also shows how uniformity varies with working distance for a given surface size. At surface sizes below 100 cm^2^, uniformity is lowest at the furthest working distance, 50 mm, whereas for surface sizes above 100 cm^2^ uniformity is lowest at the closest working distance, 10 mm. Given that the lamp emitting surface is roughly 100 cm^2^, the larger surface sizes (121 and 144 cm^2^) will capture more photons at their edges as the working distance increases, thus increasing uniformity. In the case of surface sizes below 100 cm^2^, short working distances will give an illumination pattern similar at the center of the surface to the edges, thus highly uniform illumination is observed.

Given that power plateaus for surfaces above 100 cm^2^ and uniformity is high for surfaces below 64 cm^2^, the ideal surface size lies somewhere between 64 cm^2^ and 100 cm^2^ for a balance of the two measured criteria. The power of the lamp can be increased linearly simply by increasing the current delivered to the LED (see Fig. S4), whereas the uniformity can only be increased by redesign of the lamp's optics, therefore the 64 cm^2^ surface will be chosen in this case to carry forward to further characterize the lamp and conduct discrete ordinate method simulations.

### 3.2 Incident radiation vs working distance

**Fig. 6:**
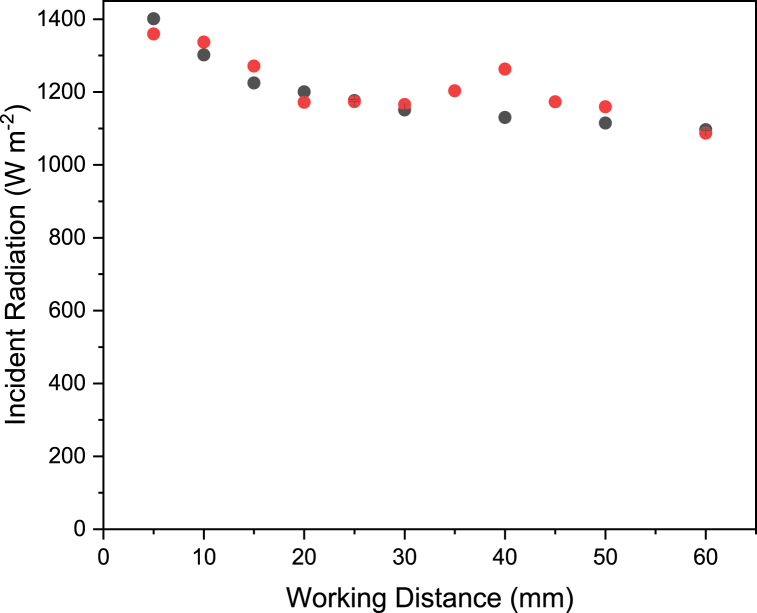
Peak incident radiation versus working distance measured by radiometry (black squares) and from discrete ordinate method (red circles) for a target surface of 64 cm^2^.

Fig. 6 shows the peak incident radiation measured by DOM and radiometry at a range of working distances. Both DOM and radiometry methods highlight decreasing peak incident radiation with increasing working distance from the lamp, as would be expected. The two methods show good agreement (±10%), except for at a working distance of 40 mm, where DOM gives a value of 1263 W m^-2^, whereas radiometry measurements at this distance give a value of 1130 W m^−2^. The increase in peak incident radiation from 30 to 40 mm for the result from the DOM model can be explained by constructive interference between individual LED fields. In the case of the physical lamp's field measured by radiometry, the extent of constructive interference at a working distance of 40 mm may not be the same due to differences in physical parameters such as refractive index or scattering coefficient, as well as scan averaging from the radiometer smoothing out the peaks and troughs created by constructive interference. Equally, subtle differences in the designed and manufactured lamp could impact this value. The DOM point at 40 mm is not anomalous, as the trend is confirmed by incident radiation vs working distance DOM simulations at 35 and 45 mm.

### 3.3 Power vs working distance

**Fig. 7:**
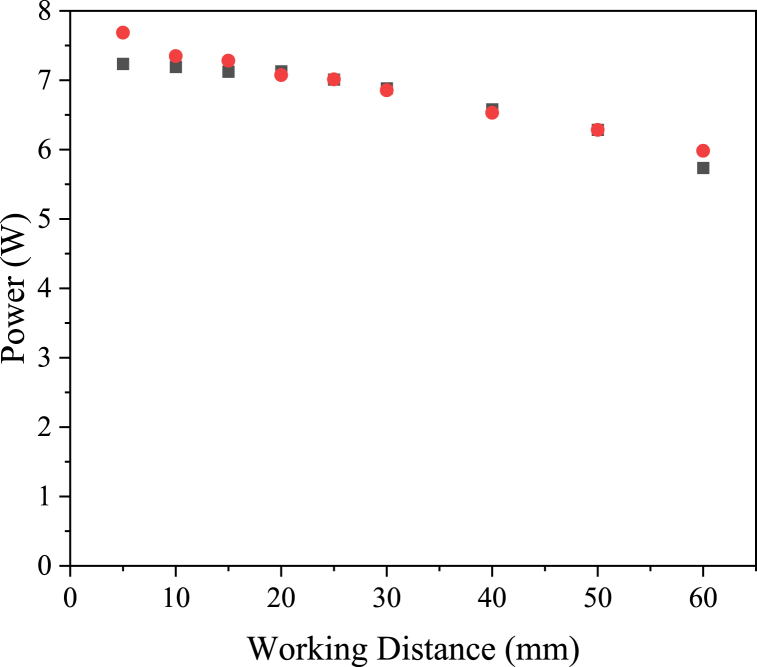
Power (W) on a 64 cm^2^ surface, measured by radiometry (red circles) and calculated with discrete ordinate method (black squares).

The power measured by DOM and radiometry show good agreement (±10%) across the range of working distances, as shown in Fig. 7. In the case of DOM, the value is calculated by taking the average absorbed incident radiation acting on the target surface and multiplying by the area of the target surface (64 cm^2^), whilst for the value calculated from radiometry, all values recorded in the target area are summated and then divided by the number of points per cm^2^. The power measurements are less sensitive to individual irradiance values than the peak incident radiation values in Fig. 6, which contributes to the better agreement between DOM and radiometry for power measurements.

**Fig. 8:**
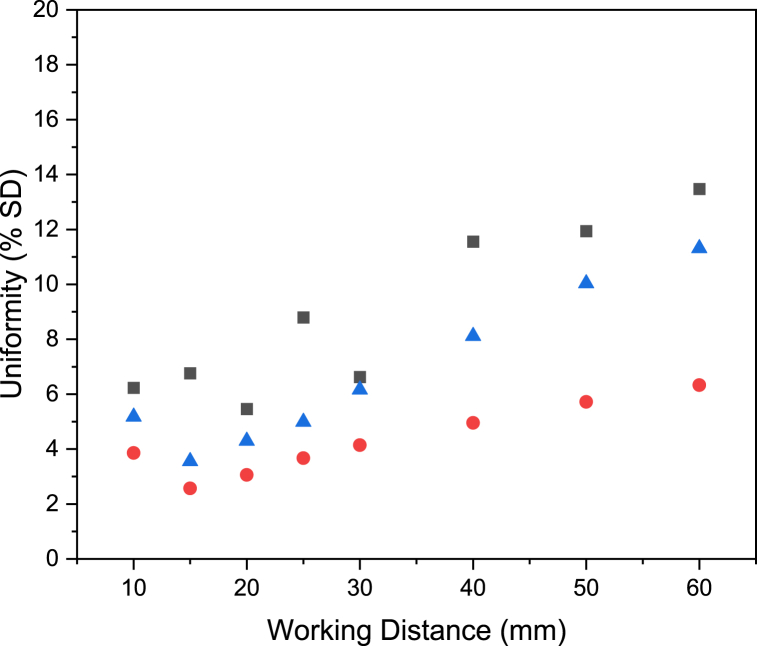
Surface uniformity (% SD) calculated from maximum horizontal incident radiation scans (red circles), full radiometry scans (blue triangles), and DOM simulations (black squares).

### 3.4 Uniformity vs working distance

The uniformity of irradiation on the target surface is calculated using Eq. (3), where percentage standard deviation is being used to represent uniformity. In this case, low standard deviation of incident radiometry measurements on the surface indicates highly uniform illumination. The uniformity over a range of working distances is shown in Fig. 8. The uniformity from DOM and radiometry techniques shows a minimum percentage standard deviation at 20 and 15 mm working distances respectively from the lamp for a 64 cm^2^ target surface. There is also consistently greater variance from the DOM data compared to the radiometry data. The radiometry measurements exhibit higher uniformity due to scan averaging, scan speed, and the distance between measurements (scan resolution). The radiometry scan averaging time of 1 s coupled with a scan speed of 10 mm s^−1^ allows for scans to be completed in a reasonable timeframe but at the expense of peaks and troughs lost through averaging. Similarly, measuring fewer points from scanning radiometry allows for faster scan times but at the expense of highly granular data. A further consideration is the aperture size of the radiometer, which in this study is 8 mm. A radiometer measures the average irradiance over the detector surface, whereas DOM simulation measures the local irradiance, therefore the simulated data will identify maximum and minimums not detected by radiometry [13].

**Fig. 9:**
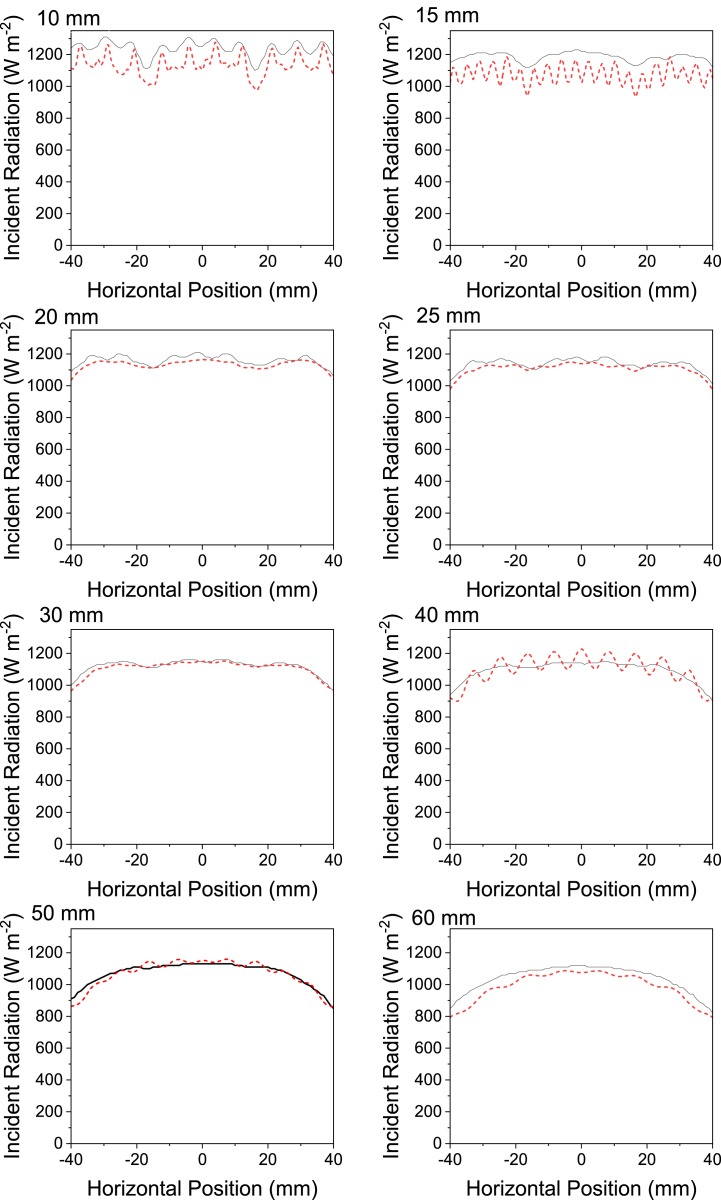
Maximum horizontal incident radiation plots from radiometry scans (black solid line) and discrete ordinate method simulations (red dashed line) at working distances from 10 mm to 60 mm.

This is best demonstrated by maximum horizontal incident radiation plots, shown in Fig. 9, and surface incidence radiation plots, shown in Fig. S5. The maximum horizontal incident radiation plots, particularly at working distances of 40 and 15 mm, highlight the individual fields and constructive interference from the LED in the lamp with DOM simulations, where the plot oscillates, whereas for radiometry data the plots are much smoother due to scan averaging. Additionally, the resolution of the two scan methods plays an important role. In the case of maximum horizontal intensity plots from DOM simulations, the plots are based on 190 data points, whereas the radiometry scans collected 81 data points. The greater resolution scans from DOM simulations allow for the constructive interference to be identified here but not from radiometry data. The differences in resolution between radiometry and simulation methods can also be observed in Keshavarzfathy et al.‘s work, where a UV LED photoreactor was measured by a radiometer through the center line of a photreactor and the results compared to the solution of the RTE in ANSYS Fluent [13]. In this paper, the radiometry data points match well to those from the simulation results, although radiometry does not necessarily identify the maximum and minimum irradiance values in the measured field. The standard deviation at 30 mm working distance calculated from DOM data is very close to the radiometry scan data variance. The smooth surface measured at 30 mm by radiometry is less a result of scan averaging and more likely a true reflection of the radiation pattern. In the case of the surface incident radiation plots in Fig. S3, the irradiance from individual LED can be seen in the plots for DOM simulations at 10 mm and 15 mm working distances. At 40 mm, the surface incidence radiation plots for DOM simulations have individual peaks not measured by scanning radiometry.

It is also important to consider the angle of acceptance of the radiometer used in this paper. It has been shown in literature that the Ophir PD-300 R M-8W has an angle of acceptance of 114.5°, which is considered high for radiometers [22]. As a result of this high angle of acceptance, the radiometer will be measuring incident radiation from across the lamp surface at short working distances. At longer working distances, the light is mainly coming from directly in front of the radiometer. This means there may be a slight averaging effect of incident radiation measurements close to the lamp face. As a result, the standard deviation within a scan will be lower at shorter working distances, leading to a shift of the ideal working distance to shorter distances which could explain the disagreement in ideal working distances between radiometry techniques and DOM.

In the context of surface illumination, power and uniformity are more important design parameters than localized incident radiation on the surface. Fig. 7, Fig. 8, Fig. 9 highlight that DOM and radiometry show good agreement for power and uniformity measurements, suggesting that DOM simulations could be used as a fast and reliable alternative scanning radiometry in lamp design for surface illumination.

The fact that all three measurement techniques used in this paper can reliably be used to determine the ideal working distance for even illumination is encouraging for applications beyond photocatalysis that require it, such as dentistry [24,25], horticulture [26], and adhesive curing [27]. Given that the maximum horizontal incident radiation scans suggest a minimum standard deviation at 15 mm from the lamp, in agreement with the full radiometry scan, this simple, single axis technique could be preferentially used, at least for lamps with a high degree of symmetry. Furthermore, discrete ordinate method simulations have given similar values to the full radiometry scan for power measurements, peak incident radiation, and uniformity. One particularly useful application of DOM in the context of uniformity would be for scaled up systems requiring several lamps. With validation complete on a single lamp, a system that requires multiple identical lamps could be assembled and simulated using DOM to determine an arrangement suitable to the target surface.

### 3.5 Impact of individual LED spectrum on uniformity

**Fig. 10:**
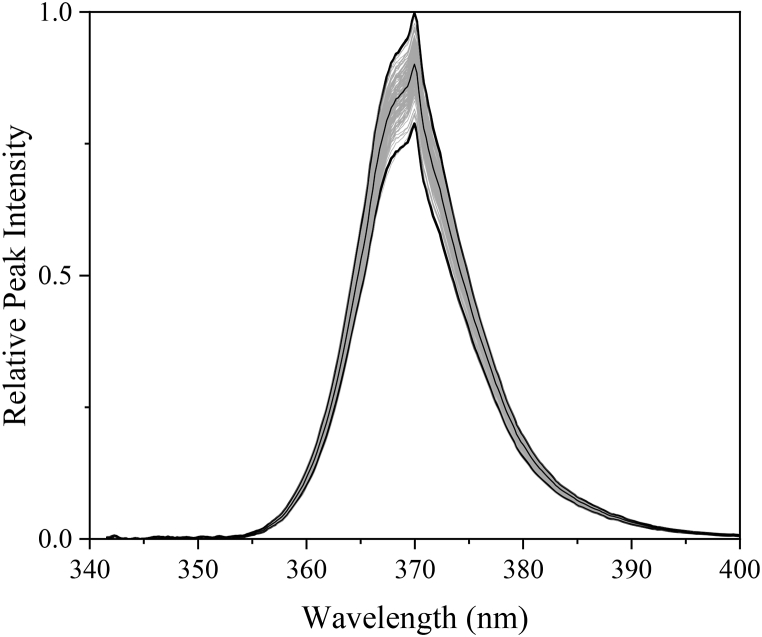
Spectral measurements of 144 LED from the UVA LED lamp. Maximum, minimum, and average spectral lines represented by black lines.

Whilst optical design, target surface size, and working distance all impact the uniformity of illumination from a UV lamp, another contributing factor is the output power and spectrum of each individual LED in the lamp. Fig. 10 shows the peak wavelength for all 144 LED occurs at 369.76 nm (for the first 60 s after starting the lamp, the wavelength is 367.4 nm, before shifting to 369.76 nm, see Fig. S3), whilst peak luminous intensity ranges by ±12% from the average. The specification provided by Seoul Viosys for the LED used in this lamp states that the luminous intensity will be in the range of ±10% [21]. Accounting for variations in the lamp assembly process would suggest that the LED are within specification. The area under the curve of each plot, representing the total power from each LED, sees a similar range to wavelength peak, with ±12.4%. In near-field applications, these results will have some significance with respect to uniformity, as the individual power output from one LED will be localized to the area directly in front of it. In far-field applications, the individual power output will be less significant to uniformity due to constructive interference between neighboring LED.

## 4 Conclusions

A technique to determine the ideal working distance of an LED lamp from flat target surface for the maximum spatial uniformity using radiometry and discrete ordinate method modelling on ANSYS Fluent has been demonstrated. This paper has shown that DOM simulations suggest an ideal working distance of 20 mm between an LED lamp and a 64 cm^2^ surface, whilst for the same target surface size radiometry measurements give an ideal distance of 15 mm. The difference in ideal working distances found from radiometry and DOM simulations can be mainly attributed to the resolution of the respective data sets. Additionally, the scan speed of the radiometer, and the wide angle of acceptance of the radiometer contribute to differences in surface incident radiation and maximum horizontal incident radiation plots from DOM simulations and radiometry. The paper also demonstrates that full XY radiometry scans and maximum horizontal incident radiation scans conclude the same working distance for maximum uniformity. Furthermore, peak incident radiation and power were measured by discrete ordinate method and radiometry with good agreement between the results. The findings of this paper suggest DOM simulations can be used to design LED lamps quickly and reliably for highly uniform illumination on flat surfaces.

## Author contribution statement

Conor Reddick: Performed the experiments; Analysed and interpreted the data; Wrote the paper.

Cintia Casado: Conceived and designed the experiments; Analysed and interpreted the data; Wrote the paper.

Ken Reynolds, Simon Stanley, Cristina Pablos, Javier Marugán: Conceived and designed the experiments; Analysed and interpreted the data; Contributed reagents, materials, analysis tools or data; Wrote the paper.

## Data availability statement

Data will be made available on request.

## Declaration of competing interest

The authors declare that they have no known competing financial interests or personal relationships that could have appeared to influence the work reported in this paper.
