# Supplementary material for: Evaluation of the uniformity of UVA LED illumination on flat surfaces: Discrete ordinate method, single axis, and surface scanning radiometry
Source: Heliyon. 2023 May 20;9(6):e16557. doi: 10.1016/j.heliyon.2023.e16557 (PMC10220407; doi:10.1016/j.heliyon.2023.e16557)
Supplement: Multimedia component 1 [file mmc1.docx]

**Supplementary Information**

Table S1: Irradiance vs working distance scans

| Test type | Distance of lamp window from radiometer (mm) | Channel current (mA) | Scan height (mm) | Scan width (mm) | Horizontal offset (mm) | Vertical offset (mm) | Dwell time (s) | Distance between points, horizontal direction (mm) | Horizontal points | Distance between points, vertical direction (mm) | Vertical points | Vertical scan speed (mm s^-1^) |
| --- | --- | --- | --- | --- | --- | --- | --- | --- | --- | --- | --- | --- |
| Irradiance vs working distance | 10 to 60 | 250 | 25 | 25 | 27.5 | 72.5 | 1 | 2.5 | 11 | 2.5 | 11 | 10 |
| Peak irradiance vs current | 15 | 20 to 750 | 25 | 25 | 27.5 | 72.5 | 1 | 2.5 | 11 | 2.5 | 11 | 10 |
| Maximum horizontal irradiance | 10 to 60 | 250 | 0 | 150 | -35 | 85 | 0 | 1 | 151 | 0 | 0 | 10 |
| Surface irradiance | 10 to 60 | 250 | 150 | 150 | -35 | 10 | 0 | 5 | 31 | 2.5 | 61 | 10 |

Fig. S1: LED spectrum and photon energy over the wavelength range of the LED

Fig. S2: Top Left: Motor gantry in black box with radiometer attached. Top Right: Radiometer. Bottom Left: Ophir StarBright power meter. Bottom Right: Lamp in position in black box in front of motor gantry ready to be scanned.


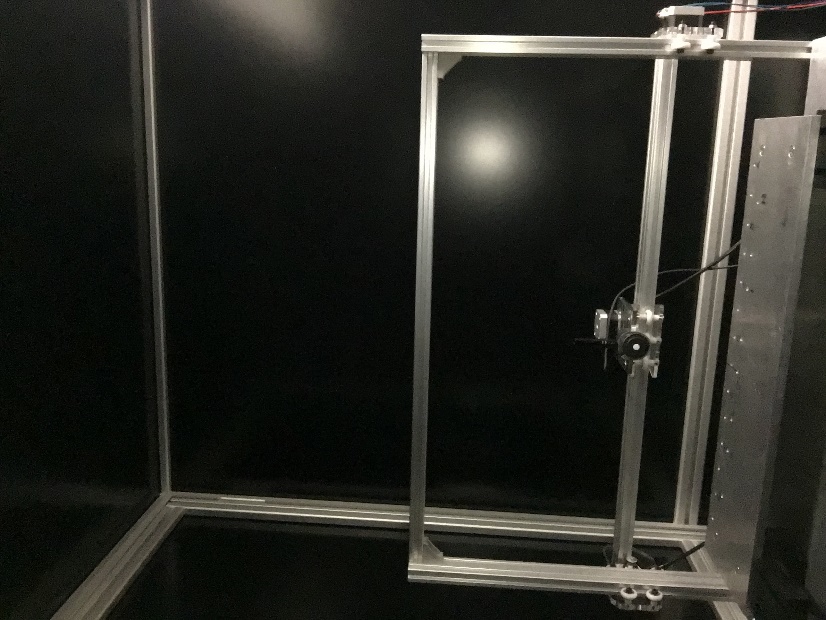

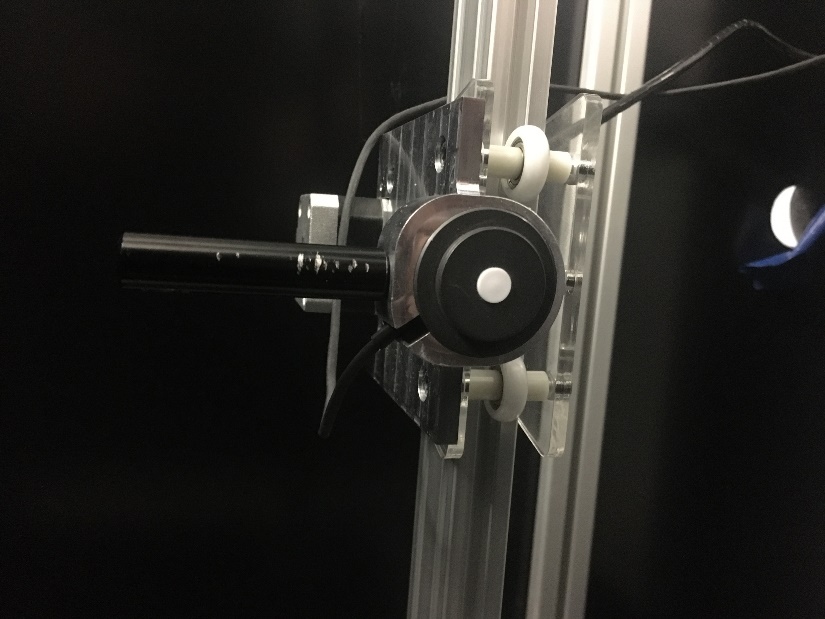

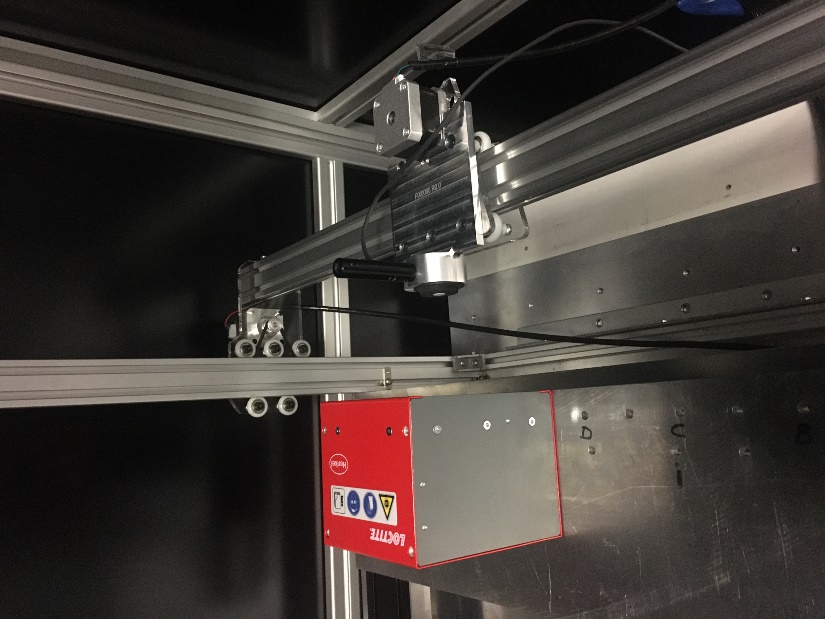

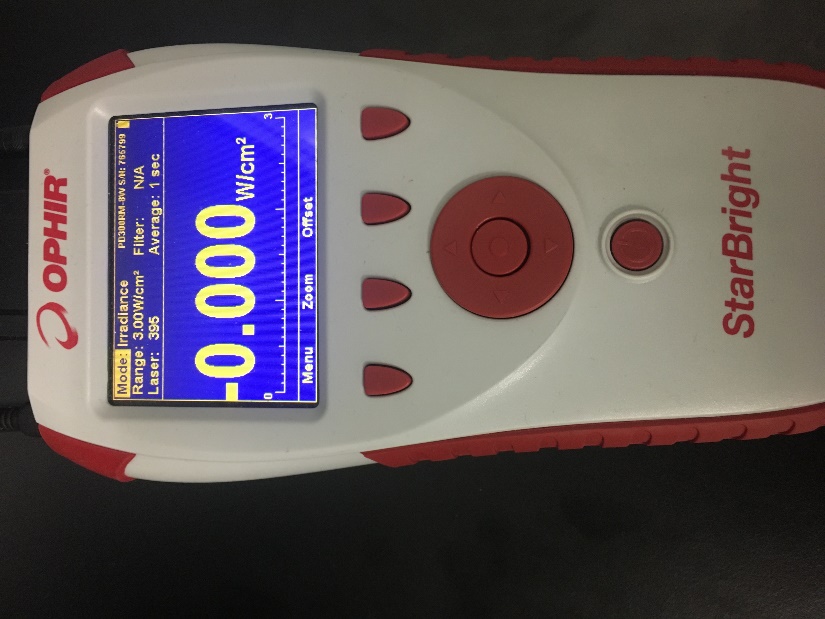

Fig. S3: Spectral shift of a UVA LED over a period of 1 h.

Fig. S4: Forward current against incident radiation measured by radiometry for the UVA LED lamp.

WD: 25 mm

WD: 20 mm

WD: 15 mm

WD: 10 mm

Radiometry DOM

Fig. S5A: Surface Incident Radiation plots created from scanning radiometry data and discrete ordinate method data. Top to bottom: 10, 15, 20, and 25 mm working distance (WD). Radiometry plots are shown on the left-hand side of each box, while discrete ordinate method plots are shown on the right-hand side.

**

WD: 60 mm

WD: 50 mm

WD: 40 mm

WD: 30 mm

Radiometry DOM

Fig. S5B: Surface Incident Radiation plots created from scanning radiometry data and discrete ordinate method data. Top to bottom: 30, 40, 50, and 60 mm working distance (WD). Radiometry plots are shown on the left-hand side of each box, while discrete ordinate method plots are shown on the right-hand side.
